# Comparison of Bone Formation After Early and Late Secondary Alveolar Bone Grafting in Patients With Unilateral and Bilateral Cleft Lip and Palate

**DOI:** 10.1002/cre2.70116

**Published:** 2025-03-18

**Authors:** Aya Maeda‐Iino, Shoko Nakagawa, Kanako Marutani, Yasuhiko Oga, Kotaro Tahakashi, Sayaka Hino, Mika Fukushima, Toshiro Kibe, Masahiro Tezuka, Norifumi Nakamura, Shouichi Miyawaki

**Affiliations:** ^1^ Field of Developmental Medicine, Health Research Course, Department of Orthodontics and Dentofacial Orthopedics Graduate School of Medical and Dental Sciences, Kagoshima University Kagoshima Japan; ^2^ Department of Orthodontics Center of Developmental Dentistry, Medical and Dental Hospital, Kagoshima University Kagoshima Japan; ^3^ Department of Oral and Maxillofacial Surgery, Field of Maxillofacial Rehabilitation, Graduate School of Medical and Dental Sciences Kagoshima University Kagoshima Japan

**Keywords:** alveolar bone grafting, orthodontic tooth movement, osteogenesis

## Abstract

**Objectives:**

Late‐stage secondary alveolar bone grafting (SBG) has a poor prognosis; however, the case selection and timing of orthodontic treatment have not been thoroughly investigated. This study aimed to clarify the prognosis of early‐SBG and late‐SBG in patients with unilateral or bilateral cleft lip and palate.

**Materials and Methods:**

Fifty‐six patients underwent early‐SBG performed before the eruption of the cleft‐adjacent lateral incisors or canines (early‐SBG group). Ten patients underwent late‐SBG requiring significant tooth movement for gap closure; SBG was performed before phase II treatment for treatment efficiency, and tooth movement began approximately 3 months post‐SBG (late‐SBG group). Pre‐SBG cleft width was measured; bone‐bridge formation using the Chelsea scale and cleft gap closure post‐SBG were evaluated. Types A and C bone bridges were considered adequate. These items were statistically compared between groups.

**Results:**

Cleft width in the early‐SBG group was significantly smaller than that in the late‐SBG group (*p* < 0.001). There were no significant differences in the percentage of adequate bone‐bridge formation (*p* = 0.055) and cleft‐gap closure (*p* = 1.000) between groups. However, the ratio of type A bone bridges was significantly higher in the early‐SBG group than in the late‐SBG group (*p* = 0.035).

**Conclusion:**

These results suggest that, even in late‐SBG, early orthodontic tooth movement to the graft bone leads to the formation of an adequate bone bridge, similar to early‐SBG. However, the maintenance of cervical grafted bone after late‐SBG may be less than that after early‐SBG.

## 1 Introduction

The gold standard for secondary alveolar bone grafting (SBG) in patients with cleft lip and palate (CLP) is between the ages of 8 and 12 years (early‐stage SBG), before the eruption of the cleft‐adjacent lateral incisors or canines and when the root has reached one‐third to two‐thirds of its final length (Bittermann et al. 2016; Waite and Kersten 1980). In contrast, late‐stage SBG, performed after the age of 13 years and after the eruption of cleft‐adjacent lateral incisors or canines, is often associated with poor graft incorporation and a significantly higher complication rate (Bittermann et al. 2020; Meyer and Mølsted 2013). However, the CLP factors, such as cleft width and the timing of orthodontic tooth movement, were not sufficiently analyzed in these reports, making it unclear whether the prognosis is affected only by the time at which SBG is performed.

When adjacent teeth erupt into grafted bone, the eruption process helps suppress bone resorption (Kalaaji et al. 1996; Jia et al. 2006; Van der Meij et al. 2001). In addition, the cleft‐adjacent central incisors or canines should be moved into the grafted bone area 3–6 months after SBG or even before 3 months to minimize resorption of the grafted bone (Åbyholm et al. 1981; Bergland et al. 1986; Nique et al. 1987; Turvey et al. 1984). Grafted bone quality after SBG in cases with a large cleft width is poorer than in cases with a small cleft width, (Maeda et al. 2014; Freihofer et al. 1993) possibly due to the difficulty in maintaining the bone owing to the absence of erupting or moving cleft‐adjacent teeth into the grafted bone.

Based on these reports and previous experiences, since 2012, we have established two protocols for SBG according to the case (Maeda‐Iino et al. 2021). In patients with a small cleft width, where the cleft‐adjacent tooth would erupt in the grafted bone and space closure could be achieved with minimal orthodontic tooth movement, SBG was performed before the eruption of the cleft‐adjacent tooth (early‐SBG protocol). In patients with a cleft requiring significant orthodontic tooth movement for space closure, SBG was performed before commencing phase II and edgewise treatments at approximately 3 months after SBG (late‐SBG protocol) to consider treatment efficiency (Maeda‐Iino et al. 2021). The prognosis of bone‐bridge formation using these protocols is unknown. Therefore, we aimed to clarify the prognosis of bone grafting using these two protocols.

## 2 Materials and Methods

### 2.1 Patients

Among consecutive patients with CLP treated at the Department of Orthodontics between 2012 and 2021, 66 patients who underwent early‐ or late‐SBG and met the exclusion and inclusion criteria were included. All patients were treated in accordance with the following treatment protocol for CLP. Presurgical orthopedic treatment using Hotz's plate or pre‐surgical nasoalveolar moulding (PNAM) was applied until primary lip repair, which was performed in accordance with a triangular flap technique at around 3–4 months of age in patients with cleft lip. Primary palatal repair was performed at around 1–1.5 years of age in accordance with pushback surgery method. According to the guidelines of the ethics review committees, patients were not required to provide informed consent for the present study because this study is retrospective. Instead, the opt‐out method of consent was applied for this study via the Kagoshima University Medical and Dental Hospital websites. The study design was approved by the Kagoshima University Ethics Committee (approval number #200310(661)‐1). The study was conducted according to the ethical principles outlined in the Declaration of Helsinki.

The inclusion criteria were as follows: (1) complete unilateral CLP (UCLP) or bilateral CLP (BCLP), (2) orthodontic and surgical treatment for CLP performed at our hospital, (3) treatment by SBG (all grafted bone was harvested from the ilium), and (4) treatment using early‐SBG or late‐SBG protocols. Exclusion criteria included the unavailability of radiographs required for the present study. Orthodontic treatment and SBG were performed under the supervision of a professor.

**FIGURE 1:**
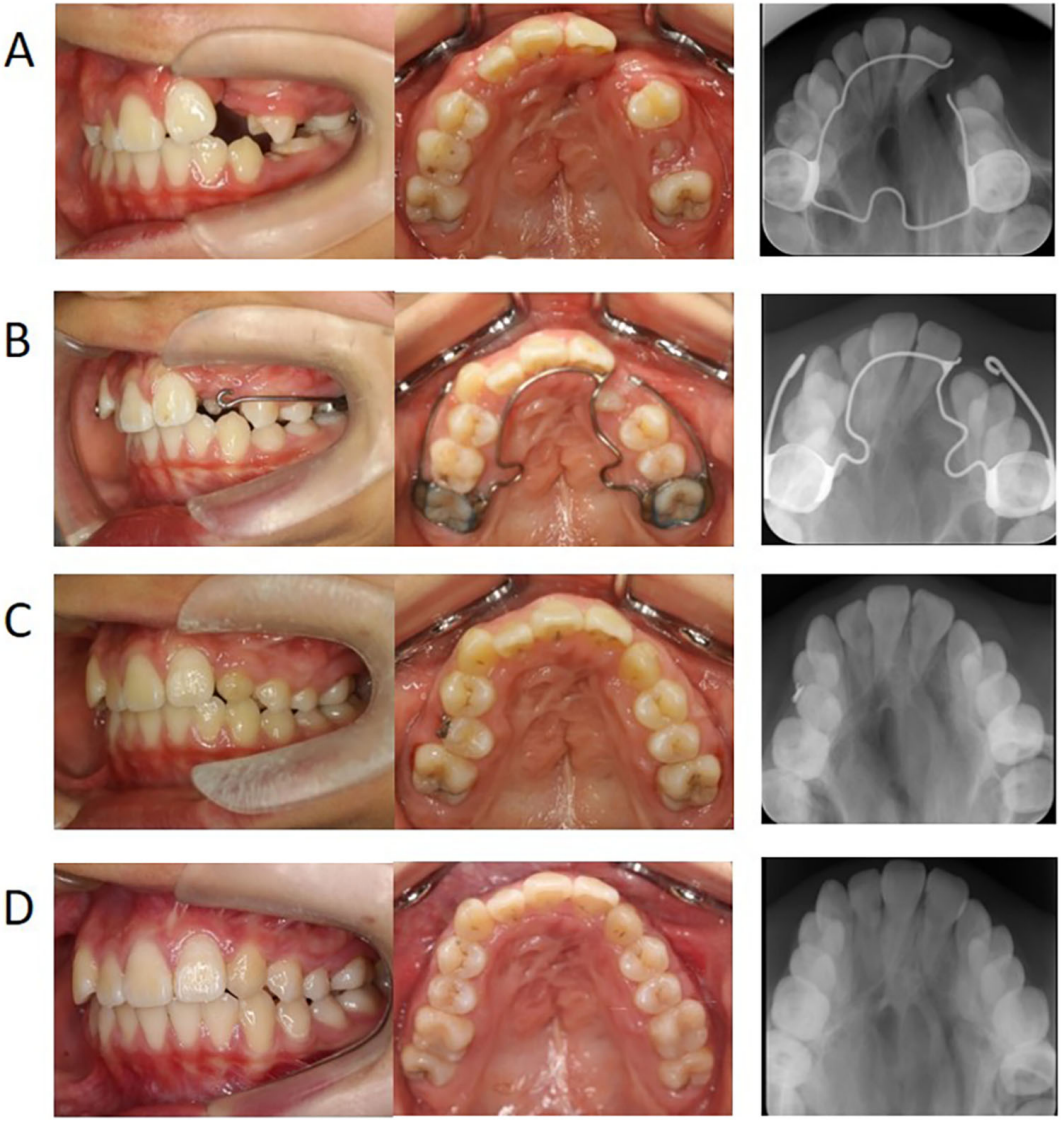
A typical case treated using the early‐SBG protocol (progress of SBG and phase I treatment after SBG). (A) pre‐SBG (age, 9 years and 10 months), (B) 12 months after SBG, improvement in the palatal eruption of the canine; (C) 2 years and 2 months after SBG; (D) 5 years and 5 months after SBG (before the start of phase II treatment). SBG, secondary bone grafting.

Cases were included in the early‐SBG group if they met the following criteria: (1) improvement in the position of the cleft‐adjacent central incisors using lingual inclination or rotation (without moving the roots into the cleft area) and expansion of the maxillary arch before SBG if the maxillary arch was not discontinuous due to segment constriction; (2) a cleft width that could be closed if the cleft‐adjacent teeth erupted into the grafted bone; (3) SBG performed before the eruption of cleft‐adjacent lateral incisors or canines after the complete eruption of cleft‐adjacent central incisors; (4) improvement in the distal inclination of cleft‐adjacent central incisors post‐SBG; and (5) minor mesial movement or improvement in crossbite after eruption of a cleft‐adjacent tooth was needed (Figure 1). Of the consecutive patients, 56 met these criteria.

**FIGURE 2:**
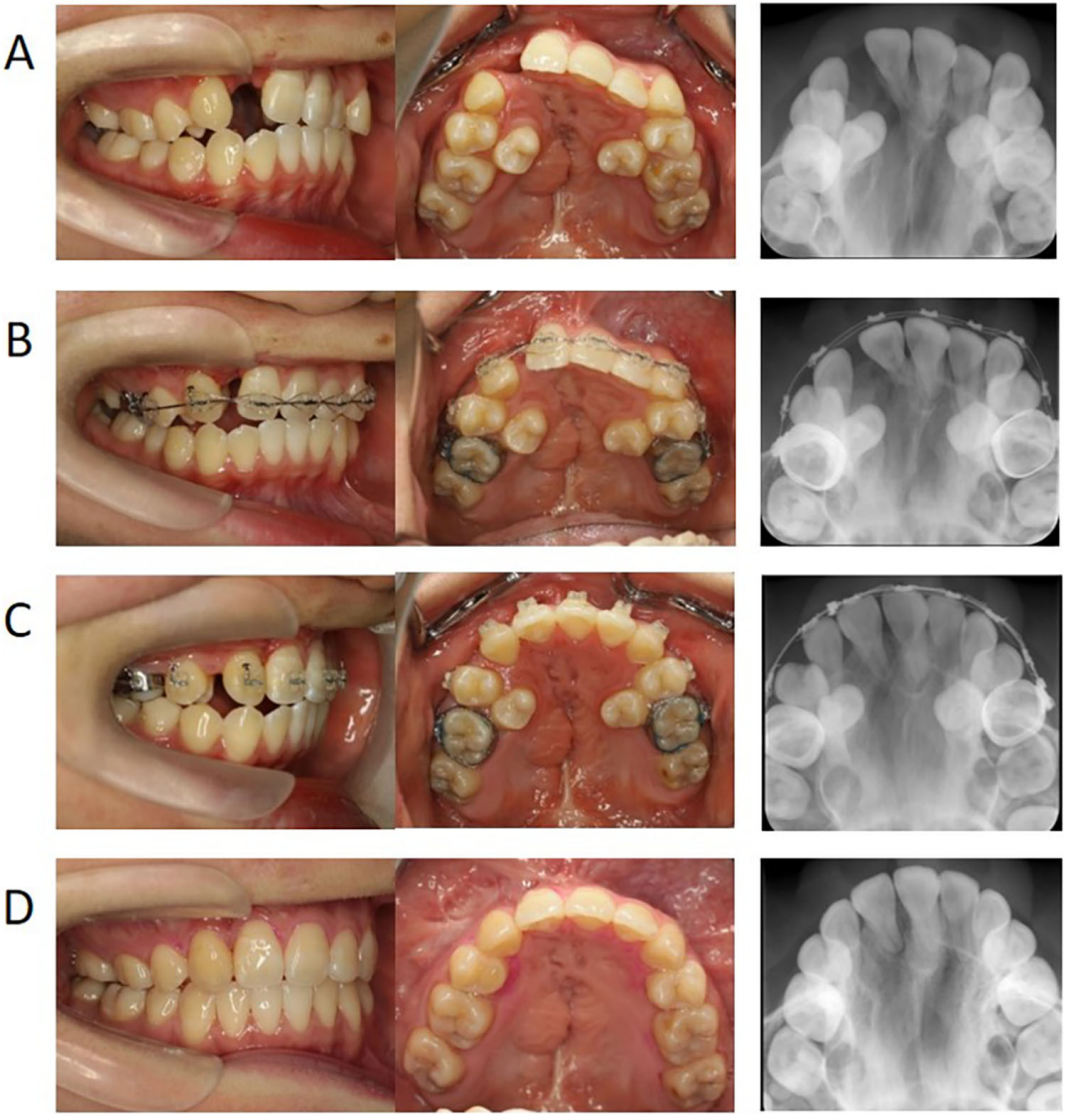
A typical case treated using the late‐SBG protocol (progress of SBG and phase II treatment). (A) pre‐SBG (age, 13 years and 9 months), (B) at the beginning of leveling of maxillary teeth 3 months after SBG; (C) 1 year and 2 months after SBG, completion of mesial movement of the maxillary canine; (D) post‐edgewise treatment. SBG, secondary bone grafting.

Cases were included in the late‐SBG group if they met the following criteria: (1) improvement in the position of the cleft‐adjacent central incisors using lingual inclination or rotation (without moving the roots into the cleft area) and expansion of the maxillary arch before SBG if the maxillary arch was not discontinuous due to segment constriction; (2) a cleft that requires significant tooth movement for gap closure (such as cases where it is difficult to close the gap by only erupting the cleft‐adjacent canine into the grafted bone and it is necessary to move the teeth using an edgewise appliance); (3) SBG performed after the eruption of cleft‐adjacent canines and just before commencing phase II treatment; and (4) initiation of orthodontic maxillary tooth movement by edgewise treatment approximately 3 months after SBG for closing the cleft (Figure 2). One case with BCLP underwent bone grafting to maintain segmental continuity and not to close the gap (right side); it was excluded as it did not meet the late‐SBG group inclusion criteria. Of the consecutive patients, 10 (11 clefts) met these criteria.

### 2.2 Cleft Width Measurement and Bone‐Bridge Evaluation on Occlusal Radiographs

The presurgical cleft width was evaluated on occlusal radiographs obtained within 3 months before SBG. The cleft width was determined by inspecting the widest point (Kubota et al. 2003). To assess intra‐examiner reproducibility and reliability of the measurements, 30 randomly selected occlusal radiographs were retraced after a minimum interval of 2 months. The evaluation of discrepancies in cleft width between the first and second (matched paired *t*‐test) measurements revealed no statistically significant differences.

**FIGURE 3:**
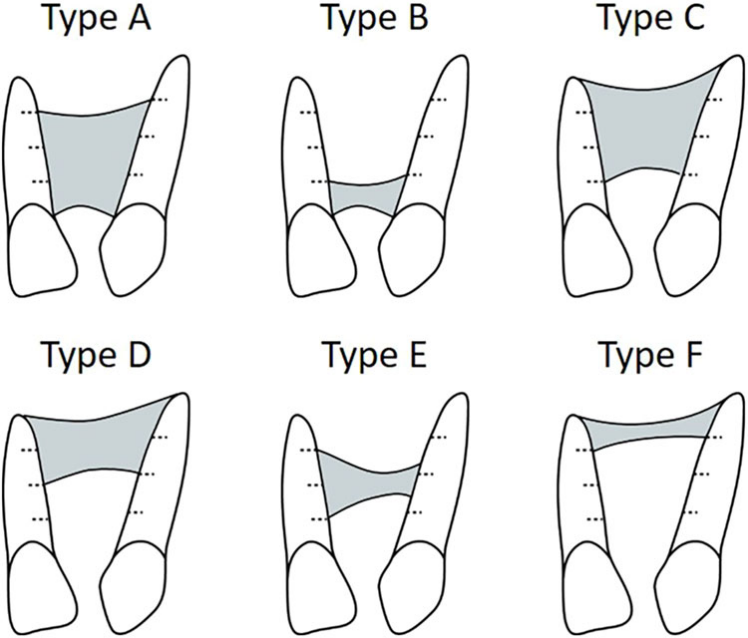
Schematic representation of the 6‐point Chelsea scale: gray parts, presence of bone tissue. Categories reflecting the position of bone relative to the cleft teeth, according to Witherow et al. (2002). Sketches of type A–D and type F bone‐bridge formation show the minimum and maximum presence of bone, respectively, in each category.

The bone bridge formed after SBG was evaluated using the Chelsea scale (Witherow et al. 2002) (Figure 3). This scale classifies bone bridges into six types:

Type A: bone tissue at the amelocemental junction with ≥ 75% of the two roots covered with bone;

Type B: bone tissue at the amelocemental junction with ≥ 25% of the two roots covered with bone;

Type C: bone tissue across ≥ 75% of the two roots from an apical direction;

Type D: bone tissue across ≥ 50% of the two roots from an apical direction;

Type E: bridge‐like bone tissue in any area of the cleft except apically and coronally; and

Type F: bone tissue across ≤ 25% of the two roots from an apical direction).

The Chelsea scale was used to evaluate the occlusal radiographs obtained approximately 12 months after SBG (average, 11.3 months after SBG, range, 6–18 months after SBG). Types A and C bone bridges were considered adequate, while other types were classified as poor bone bridges. All measurements were performed by one of the authors (A. MI.). Inter‐ and intra‐rater reliabilities of measurements for six bone bridge types (types A–F) were assessed using Kappa statistics in 30 occlusal radiographs. To assess the intra‐reliability of measurements, occlusal radiographs were re‐examined at a minimum interval of 2 months. Inter‐ (A.MI. and S.N.) and intra‐reliabilities for patient categorization into different bone‐bridge types were 0.850 and 0.963, respectively.

### 2.3 Assessment of Cleft Gap Closure After SBG

The extent of cleft gap closure after SBG was assessed by evaluating intra‐oral photographs to ensure accurate documentation of treatment progression. Closure was deemed complete when the space between the cleft‐adjacent central incisor and canine was eliminated. The timing of orthodontic tooth movement in the late‐SBG group was determined using medical records.

### 2.4 Statistical Analysis

Numerical variables (age and cleft width) were normally distributed; therefore, the significance of differences was determined using an unpaired *t*‐test. For differences in categorical variables (sex, cleft type, Chelsea scale type, and number of orthodontic space closures), significance was determined using Fisher's exact test. The observed probability was calculated for each comparison, and a *P* value of < 0.05 was considered statistically significant. Statistical tests were performed using conventional statistical analysis software (SPSS version 28.0 for Windows; SPSS Japan, Tokyo, Japan).

## 3 Results

There were no significant differences between the early‐ and late‐SBG groups in the male/female ratio and UCLP/BCLP ratio (Table 1). The cleft width before SBG in the early‐SBG group (6.47 ± 1.94 mm) was significantly smaller than that in the late‐SBG group (8.26 ± 1.92 mm; *p* = 0.006, Table 2). The age at which SBG was performed in the early‐SBG group (10.00 ± 0.80 years old) was significantly lower than that in the late‐SBG group (15.39 ± 1.17 years old; *p* < 0.001, Table 2).

**TABLE 1:** Comparison of distribution of sex and cleft type between groups.

	Early‐SBG group (*N* = 56)	Late‐SBG group (*N* = 10)	
	N	N	*p* value
Sex (male/female)	32/24	3/7	0.170
Cleft type (unilateral/bilateral)	48/8	8/2	0.641

*Note: p* value: Fisher's exact test.

Abbreviations: N, patient number; SBG, secondary bone grafting.

**TABLE 2:** Comparison of parameters between groups.

	Mean ± SD or n (number of clefts)		
Measurement or assessment	Early‐SBG group (*N* = 56, *n* = 64)	Late‐SBG group (*N* = 10, *n* = 11)	*p* value
**Assessment of cleft width**			
Age at assessment of cleft width before SBG (in years)	9.95 ± 0.80	15.33 ± 1.15	< 0.001^a^
Cleft width (mm)	6.47 ± 1.94	8.26 ± 1.92	0.006^a^
Age when SBG was performed (in years)	10.00 ± 0.80	15.39 ± 1.17	< 0.001^a^
**Assessment of bone bridge by Chelsea scale**			
Age at assessment of bony bridge formation after SBG (in years)	10.94 ± 0.85	16.34 ± 1.13	< 0.001^a^
Adequate bone bridge formation (n)	63	9	0.055^b^
Type A	59	8	0.035^b^
Type B	0	0	
Type C	4	1	
Type D	0	1	
Type E	1	0	
Type F	0	1	
Number of cleft gap closures (n)	63	11	1.000^b^

Abbreviations: N, patient number; n, number of cleft; SD, standard deviation; SBG, secondary bone grafting.

^a^ Unpaired *t*‐test.

^b^ Fisher's exact test.

**FIGURE 4:**
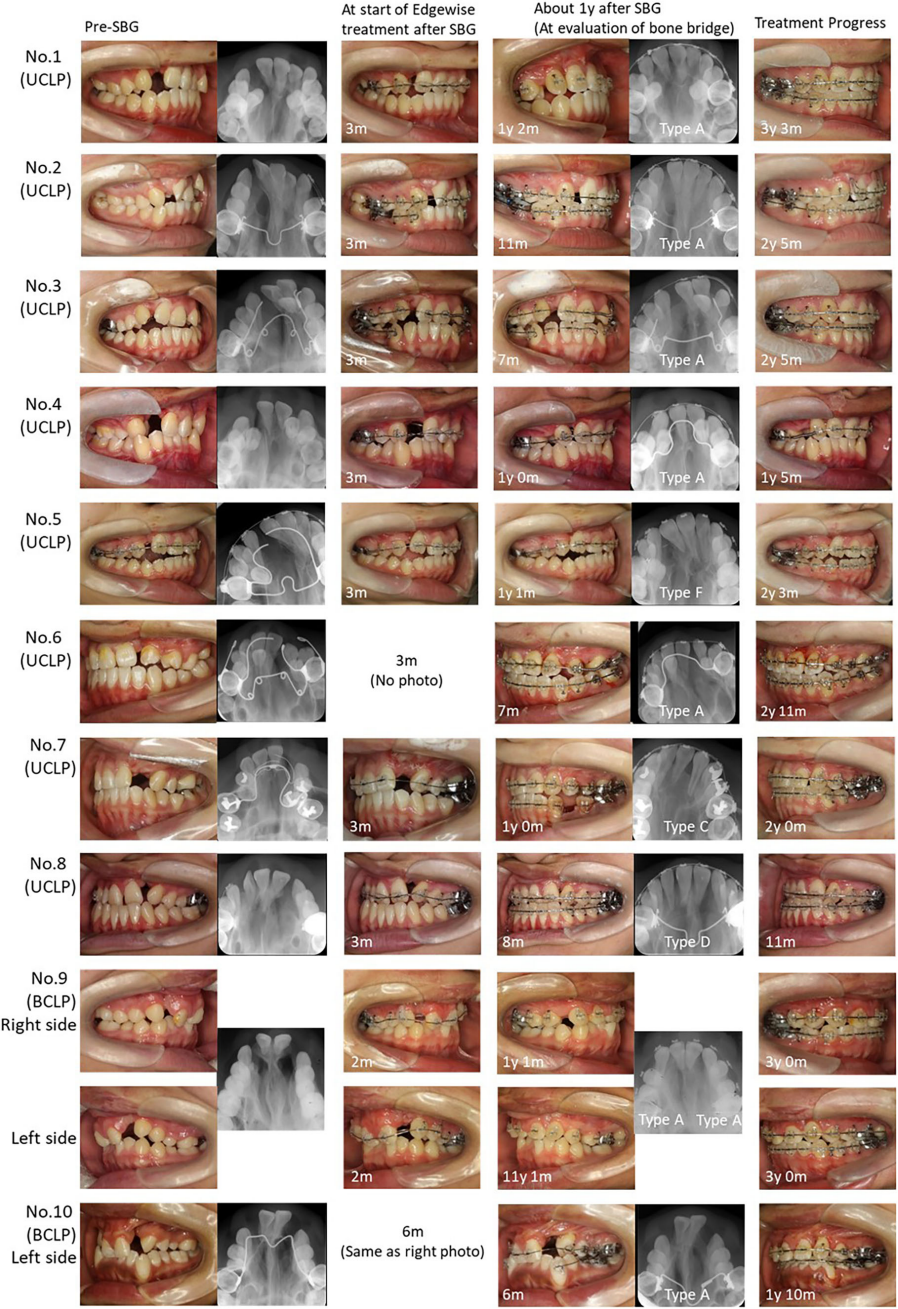
Treatment progress of all cases that underwent late‐stage SBG treatment (intraoral photographs of the cleft side and occlusal X‐rays). The figures indicate the number of years since SBG was implemented. The type of bone graft as seen on the occlusal radiograph is indicated per the Chelsea scale assessment. SBG, secondary bone grafting.

**FIGURE 5:**
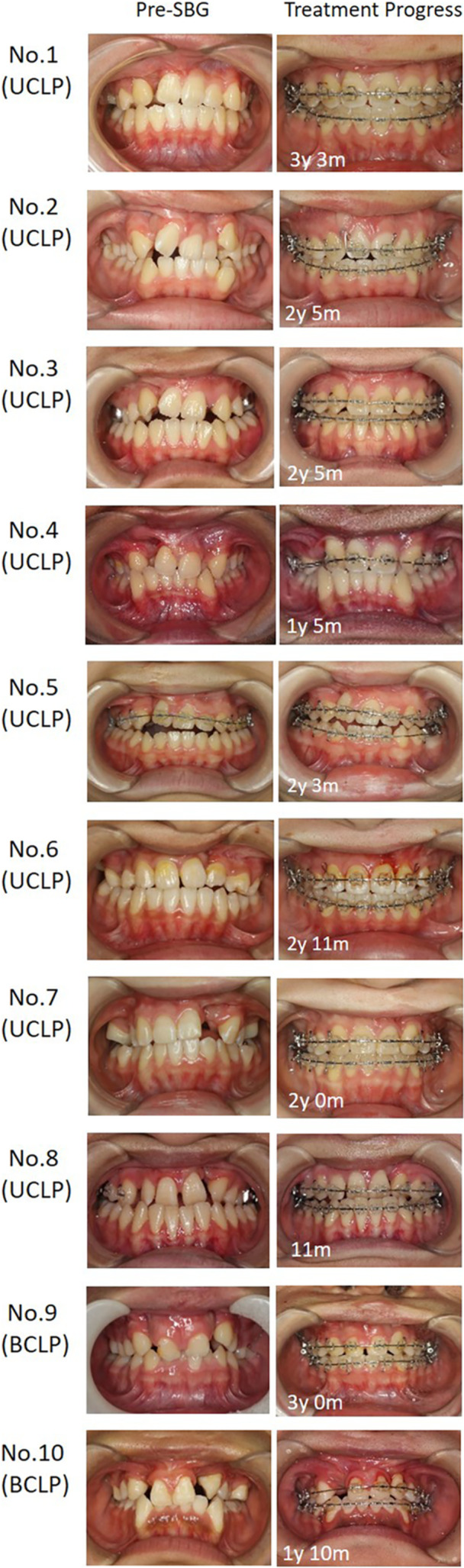
Treatment progress of all cases that underwent late‐stage SBG treatment (front intraoral photographs). The figures indicate the number of years since SBG was implemented. SBG, secondary bone grafting.

Figures 4 and 5 show the progress of phase II treatment after SBG for all cases in the late‐SBG group. There were no significant differences between the groups in adequate bone‐bridge formation (early‐SBG group 63/64 vs. late‐SBG group 9/11; *p* = 0.055, Table 2). Cleft gaps in both groups were successfully closed, except for one case in the early‐SBG group in which the lateral incisors exfoliated during SBG. There was no significant difference in the cleft gap closure ratio between the groups (early‐SBG group 63/64 vs. late‐SBG group 11/11; *P* = 1.000, Table 2).

However, the distribution of Chelsea scale types was significantly different between the groups (*p* = 0.035, Table 2). The occurrence of type A, the best bone bridge, characterized by the presence of bone tissue at the amelocemental junction with ≥ 75% of the two roots covered with bone, was higher in the early‐SBG group than that in the late‐SBG group (early‐SBG group 59/64 vs. late‐SBG group 8/11, Table 2).

## 4 Discussion

There was no significant difference in the formation of an adequate bone bridge between patients with CLP who underwent early‐stage SBG before the eruption of the cleft‐adjacent lateral incisors or canines and patients with CLP who underwent late‐stage SBG after the eruption of the cleft‐adjacent canines just before commencing phase II orthodontic treatment. However, the bone bridge formed after late‐stage SBG may be more prone to cervical bone graft loss, even if orthodontic treatment is initiated early to prevent graft bone resorption.

This study evaluated the quality of bone grafts approximately 12 months after SBG. In our hospital, occlusal radiographs are obtained approximately 3, 6, and 12 months after SBG to assess the grafted bone. In this study, we aimed to simultaneously evaluate cleft gap closure and bone‐bridge formation. Bone‐bridge formation was evaluated using occlusal radiographs obtained 12 months after bone grafting to assess the extent of gap closure. As occlusal radiographs were not obtained exactly at 12 months after SBG in any case, occlusal radiographs obtained 6–18 months after SBG were used to assess bone‐bridge formation.

Regarding the timing of SBG, the foremost consideration is dental developmental age. The current opinion is that SBG should be performed before the eruption of permanent maxillary lateral incisors or canines and after one‐third to two‐thirds root length completion; specifically, at the chronological age between 8 and 12 years (Bittermann et al. 2016; Waite and Kersten 1980). Regarding early‐stage SBG, just before the eruption of the tooth adjacent to the cleft, the tooth germ adjacent to the alveolar cleft erupts in the newly formed bone and contributes to the prevention of graft bone resorption, leading to the success of SBG (Kalaaji et al. 1996; Jia et al. 2006).

If the cleft‐adjacent lateral incisor or canine does not erupt in the grafted bone area, it would be resorbed 1 year after SBG (Van der Meij et al. 2001). When the cleft‐adjacent tooth erupts distal to the grafted bone, such as in cases with a large cleft or several missing teeth, the gap cannot be closed by the erupting cleft‐adjacent tooth alone. Therefore, resorption of grafted bone should be prevented by orthodontic tooth movement (Åbyholm et al. 1981; Bergland et al. 1986; Nique et al. 1987; Turvey et al. 1984). However, accurate prediction of timing of tooth eruption is difficult. If the grafted bone resorbs before the cleft‐adjacent tooth erupts, the tooth cannot be moved to the grafted bone, and the gap cannot be closed. In addition, because this is the mixed dentition period, only a few teeth can be used as anchorage teeth, and orthodontic movement of large teeth time consuming and inefficient. Thus, in cases wherein grafted bone would be resorbed before the eruption of cleft‐adjacent lateral incisors or canines after SBG, the cleft gap was closed without a prosthesis, and late‐stage SBG was performed after the eruption of the cleft‐adjacent canines just before commencing phase II treatment in our hospital since 2012 (Maeda‐Iino et al. 2021).

In this protocol, most of the permanent teeth, including the cleft‐adjacent teeth, erupt before SBG, which allows formulating a clear orthodontic treatment plan for the final occlusion. Additionally, in patients with wide alveolar clefts wherein it is difficult to close the gap by simply erupting the cleft‐adjacent canine into the grafted bone, it is necessary to use edgewise treatment to move the teeth during phase I treatment after early‐stage SBG; doing so may lengthen the edgewise treatment period for the entire orthodontic treatment, including phase I and II treatment, and may place a heavy burden on patients and their guardians. In such cases, edgewise treatment after late‐stage SBG has the advantage of shortening the total edgewise treatment period. However, a significantly higher complication rate and lower success rate is reported in patients who undergo late‐stage SBG (Bittermann et al. 2020; Meyer and Mølsted 2013). The study demonstrates a significant relationship between late (> 12 years) SBG in patients with BCLP and the development of complications, including wound dehiscence, oronasal fistulas, total alveolar bone graft loss, and avascular necrosis of the premaxilla (Bittermann et al. 2020). In a study of patients with CLP with a mean follow‐up of 16 years (range, 10.2–22.7 years) after SBG, the　mean age in the success group was 12.1 years compared to 13.6 years in the failure group. This study found that the success rate was significantly lower if SBG was performed after the eruption of the tooth distal to the cleft (Meyer and Mølsted 2013). Additionally, SBG is considered successful when the cleft‐adjacent tooth erupts in the newly formed bone and contributes to reduced graft bone resorption (Kalaaji et al. 1996; Jia et al. 2006). However, late‐stage SBG is performed after the cleft‐adjacent canine erupts, and therefore, this phenomenon is not expected. Because late‐stage SBG has these disadvantages, patients and their guardians should be fully informed. Orthodontists and surgeons discuss the appropriate timing of SBG before the eruption of cleft‐adjacent lateral incisors or canines. When suggesting late‐stage SBG, the risks and benefits are explained to the patient to determine the timing of the final SBG; ultimately, the patient and parents choose the timing of SBG.

The survival of the cleft‐side lateral incisor is poor (Vandersluis et al. 2020; Cassolato et al. 2009; Tereza et al. 2018). Some patients who had undergone late‐SBG had lateral incisors and the canines, although the lateral incisors erupted on the palatal side. The thin roots of the lateral incisors render their survival poor with large tooth movements. Therefore, in the late‐SBG protocol, canines were used to close large gaps. It should be noted that all outcomes in late‐SBG involve the mesial movement of the canines.

In late‐stage SBG, orthodontic tooth movement suppresses the resorption of the graft bone (Maeda et al. 2014; Maeda‐Iino et al. 2021; Dempf et al. 2002; Honma et al. 1999). Generally, the cleft‐adjacent teeth are moved in the direction of the grafted bone 3–6 months after the bone‐grafting procedure (Åbyholm et al. 1981; Bergland et al. 1986; Nique et al. 1987; Turvey et al. 1984). However, Schultze‐Mosgau et al. recommended moving the cleft‐adjacent canine into the grafted bone for space closure with orthodontic treatment as early as 4–6 weeks after SBG (Schultze‐Mosgau et al. 2003). Dempf et al. contended that the reason for lower resorption rates after orthodontic space closure is the stress introduced in the bone by the inward movement of the canine from a lateral position (Dempf et al. 2002). In late‐SBG, to minimize graft bone resorption, cleft‐adjacent canines were moved into the grafted bone 2–3 months after SBG in our hospital. In one case, tooth movement was initiated after 6 months owing to difficulty in healing and scheduling visits. In this study, there was no statistical difference in the percentage of adequate bony bridge formation between the two protocols, with early‐SBG showing a higher probability of 98.4% and late‐SBG of 81.8% (total of 96.0%). In a previous study, before using these protocols, the ratio of adequate bone‐bridge formation 6–12 months after SBG was approximately 72.1% (Maeda et al. 2014). Conventionally, SBG was performed between 8 and 12 years of age; however, the timing and amount of expansion of the maxillary segment, canine eruption, possibility of tooth eruption in the graft bone, and timing of orthodontic tooth movement were not considered. These results suggest that, similar to the eruption of tooth germs in the graft bone, the migration of teeth in the graft bone during orthodontic treatment, even after late‐stage SBG, suppresses graft bone resorption and contributes to adequate bone‐bridge formation.

Cases with a large cleft width exhibit a low success rate of SBG (Maeda et al. 2014; Freihofer et al. 1993). If the maxillary arch is not discontinuous due to segment contraction, minimal widening is performed before the SBG. Cases that underwent SBG before the eruption of the cleft‐adjacent teeth revealed a smaller cleft width compared to those that underwent late‐SBG. There was no statistical difference in the percentage of adequate bony bridge formation between early‐SBG and late‐SBG; however, the occurrence of Type A bone bridges, the best type of bone bridge after early‐SBG, was significantly higher after early‐SBG than that after late‐SBG. If the tooth germ is expected to erupt in the grafted bone and form a bone bridge, the optimal timing for SBG is just before the eruption of the cleft‐adjacent tooth, as long suggested. Many of our patients underwent SBG during this period. The most important factors that increase the success rate of bone grafting at this stage are cleft‐adjacent tooth root formation and cleft width. It is important for orthodontists to minimize maxillary arch expansion. Performing late‐stage SBG and starting tooth movement to the grafted bone early should only be performed in anticipatory cases where it is difficult to close the gap solely by the eruption of the tooth germ, and where the grafted bone may resorb. In cases with large clefts, early orthodontic tooth movement into the graft bone after late‐SBG can lead to adequate bone‐bridge formation, whereas the occurrence of type A bone bridge was lower compared to cases wherein the tooth erupted into the grafted bone. Three late‐stage SBG cases showed type C, D, or F bone bridges with at least cervical graft bone resorption than a type A bone bridge. This indicates that achieving parallelism of cleft‐adjacent teeth is difficult. However, late‐stage SBG also has advantages, such as potentially establishing occlusion without a prosthesis.

Late‐stage SBG is beneficial for dental implant placement, improving soft tissue symmetry and providing a platform for achieving adequate facial esthetics and nasal prominence in patients with CLP (Mahajan et al. 2017; Kim et al. 2012). Even if SBG cannot be performed at the recommended time—such as before the eruption of adjacent lateral incisors or canines—owing to economic reasons or difficulties in scheduling hospital visits, late‐stage SBG would still be beneficial for patients with CLP. Our results indicate that although the treatment outcomes of late‐stage SBG may be slightly inferior to that of early‐stage SBG, large tooth movements to fill the gap can still be efficiently performed in the second stage of treatment. Thus, even if patients are unable to undergo early‐stage SBG before the eruption of the canine owing to various reasons (such as, geographical circumstances, economic issues, and current restrictions on visitation due to the recent pandemic), late bone grafting can still provide a certain level of treatment quality by starting tooth movement early in phase II treatment. However, late‐ stage SBG should not be indicated for cases where eruption of the teeth into the alveolar cleft can achieve optimal space closure. The appropriate timing for SBG should be selected, depending on individual cases.

The main limitations of this study were the small sample size of patients who underwent late‐SBG and the lack of 3D evaluation by cone‐beam computed tomography (CBCT) for grafted bone. Because this was a retrospective study and early bone grafting has generally been recommended over late bone grafting, the number of patients undergoing late SBG was inevitably small. However, ours is one of the few studies with a clear treatment protocol that evaluated bone‐bridge formation after late stage SBG, and therefore, it is of vital clinical significance. To evaluate the grafted bone, CBCT has been frequently used in treatment planning for SBG in patients with CLP (Jabbari et al. 2018; Hamada et al. 2005). While this technique provides more precise information about bone morphology, the roots of the cleft‐adjacent teeth, and the grafted bone after SBG, its routine use is limited by radiation exposure and cost (Jabbari et al. 2018). In this study, bone height was evaluated at approximately 12 months after SBG in most cases, as cleft gap closure by orthodontic tooth movement is typically achieved in this time period and the grafted bone condition is expected to remain stable owing to tooth movement. Since 2015, our hospital has used CBCT before SBG and 6 months after SBG to evaluate the preoperative morphology, bone graft amount, and postoperative bone‐bridge status. Therefore, CBCT imaging was not used to evaluate the grafted bone at 12 months after SBG in this study. In a previous study that compared occlusal radiographs and CBCT for evaluation of bone height, no difference was reported in the Bergland index generated from scoring the alveolar bone height on occlusal radiographs compared to the equivalent index on CBCT (Jabbari et al. 2018). Therefore, in this study, the occlusal radiographs were used to evaluate the grafted bone instead of CBCT. We believe that conventional occlusal radiographs are sufficient for clinical follow‐up to assess postoperative bone height; however, if available, CBCT images can provide a more accurate assessment.

## 5 Conclusions

There were no significant differences in the percentage of adequate bone‐bridge formation between patients with CLP who underwent SBG before the eruption of cleft‐adjacent lateral incisors or canines and patients with CLP who underwent late‐SBG after the eruption of cleft‐adjacent canines before commencing phase II treatment. This result suggests that, even in late stage SBG, early orthodontic tooth movement to the graft bone leads to the formation of an adequate bone bridge.

In this study, patients who underwent late stage SBG had larger cleft widths and orthodontic treatment was started early in these patients to prevent graft bone resorption. However, the maintenance of cervical grafted bone may be less than that in cases with a small cleft that underwent SBG before the eruption of cleft‐adjacent lateral incisors or canines.

## Author Contributions

A.M.‐I. contributed to the study conception and design, data acquisition, analysis, and interpretation, and drafted the manuscript; S.N. and K.M. contributed to the data acquisition, analysis, and interpretation, and drafted the manuscript; Y.O., K.T., S.H, M.F., T.K, and M.T. contributed to the data acquisition and interpretation, and drafted the manuscript; N.N. contributed to the data acquisition and interpretation, and drafted the manuscript; S.M. contributed to the study conception and design and drafted the manuscript.

## Ethics Statement

The study design was approved by the Kagoshima University Ethics Committee (approval number #200310(661)‐1) and was conducted according to the ethical principles outlined in the Declaration of Helsinki.

## Consent

The opt‐out method of consent was used for this study on the web sites of Kagoshima University Medical and Dental Hospital.

## Conflicts of Interest

The authors declare no conflicts of interest.

## Data Availability

The datasets generated and analyzed during the current study are not publicly available due to ethical reasons but are available from the corresponding author on reasonable request.
